# Supplementary material for: C9orf72-G4C2 Intermediate Repeats and Parkinson’s Disease; A Data-Driven Hypothesis
Source: Genes (Basel). 2021 Aug 5;12(8):1210. doi: 10.3390/genes12081210 (PMC8391122; doi:10.3390/genes12081210)

Figure S1. Number of alleles observed for each *C9orf72*-G_4C_2-repeat-size with risk or non-risk haplotypes in the Ashkenazi Jews (PDs and controls).

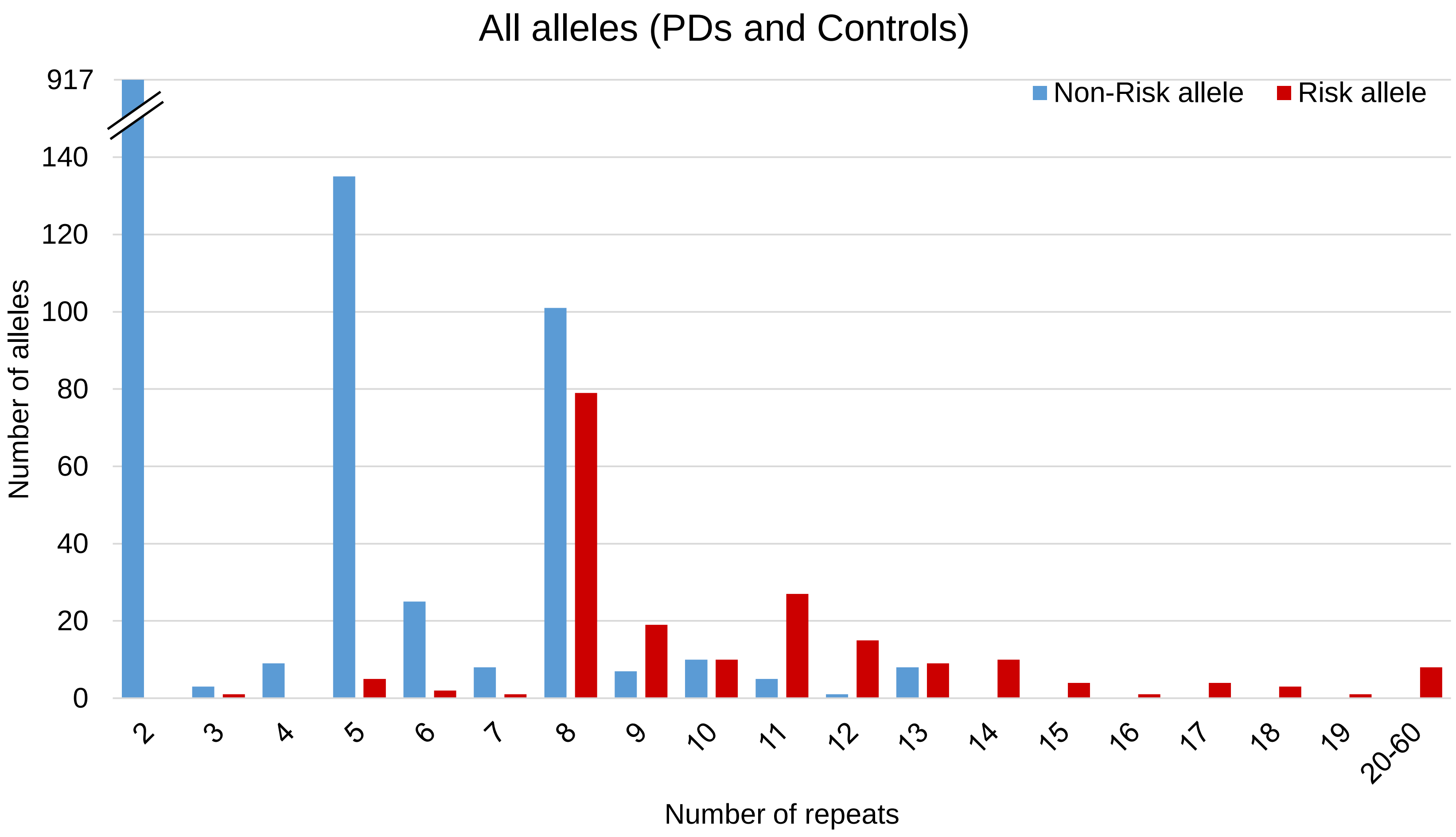

Supplement: Supplementary file 1 [file genes-12-01210-s001.zip › supplymentary date/Kobo et al_C9orf72_ms_Figure_S1_June_28_2021.pdf]
