# Supplementary material for: C9orf72-G4C2 Intermediate Repeats and Parkinson’s Disease; A Data-Driven Hypothesis
Source: Genes (Basel). 2021 Aug 5;12(8):1210. doi: 10.3390/genes12081210 (PMC8391122; doi:10.3390/genes12081210)
Supplement: Supplementary file 1 [file genes-12-01210-s001.zip › supplymentary date/Kobo et al_C9orf72_ms_Table_S1_June_28_2021.pdf]

Table S1. Allele frequencies of G_4C_2 hexanucleotide repeat lengths in *C9orf72* in Parkinson's disease patients of Ashkenazi Jewish origin and controls.

	**PD patients**		**Control** [c]
	**Non-carriers** [a]	**Carriers** [b]	
**Number of repeats**	**Frequency percent**		
2	63.1	66.2	66.4
3	0.3	0.1	0.6
4	0.4	1.2	0.3
5	9.7	8.9	10.2
6	2.5	1.7	2.0
7	0.8	0.4	0.8
8	12.7	12.1	11.0
9	1.8	1.4	1.5
10	1.6	1.2	1.5
11	2.0	2.4	2.4
12	1.0	1.2	1.0
13	1.5	1.2	0.9
14	0.7	0.9	0.3
15	0.1	0.0	0.1
16	0.1	0.0	0.3
17	0.2	0.5	0.3
18	0.2	0.0	0.1
19	0.0	0.1	0.2
20	0.2	0.0	0.0
21	0.2	0.3	0.0
22	0.1	0.0	0.1
23	0.1	0.0	0.1
24	0.1	0.0	0.0
27	0.0	0.0	0.0
28	0.0	0.1	0.0
29	0.1	0.0	0.0
32	0.1	0.0	0.0
50	0.0	0.0	0.1
58	0.1	0.0	0.0
>145	0.3	0.1	0.1
Total	100.0	100.0	100.0

[a] Patients without the *LRRK2*, *GBA* or *SMPD1* mutations.
[b] Carriers of one or more of the following mutations: *LRRK2* p.*G2019S*; *GBA* mutations: *c.84insG*, *IVS2+1G>A*, p.*V394L*, p.*N370S*, p.*L444P*, p.*R496H*, *p.E326K*, *p.T369M*, *p.R44C* and Rec370; *SMPD1* p.*L302P*.
[c] The cohort of controls has been recently published [26].
